# Blood and sputum eosinophils in COPD; relationship with bacterial load

**DOI:** 10.1186/s12931-017-0570-5

**Published:** 2017-05-08

**Authors:** Umme Kolsum, Gavin C. Donaldson, Richa Singh, Bethan L. Barker, Vandana Gupta, Leena George, Adam J. Webb, Sarah Thurston, Anthony J Brookes, Timothy D. McHugh, Jadwiga A. Wedzicha, Christopher E. Brightling, Dave Singh

**Affiliations:** 1 Division of Infection, Immunity and Respiratory Medicine, School of Biological Sciences, Faculty of Biology, Medicine and Health, Manchester Academic Health Science Centre, The University of Manchester and University Hospital of South Manchester NHS Foundation Trust, Manchester, M23 9QZ UK; 2 The Medicines Evaluation Unit, Manchester, M23 9QZ UK; 3 Department of Infection, Immunity, and Inflammation, Institute for Lung Health, NIHR Respiratory Biomedical Research Unit, University of Leicester, Leicester, UK; 4 Department of Genetics, University of Leicester, Leicester, UK; 5 Centre for Clinical Microbiology, University College London, London, UK

**Keywords:** Pulmonary eosinophilia, COPD exacerbations, Bacterial infections

## Abstract

**Background:**

Sputum and blood eosinophil counts predict corticosteroid effects in COPD patients. Bacterial infection causes increased airway neutrophilic inflammation. The relationship of eosinophil counts with airway bacterial load in COPD patients is uncertain. We tested the hypothesis that bacterial load and eosinophil counts are inversely related.

**Methods:**

COPD patients were seen at stable state and exacerbation onset. Sputum was processed for quantitative polymerase chain reaction detection of the potentially pathogenic microorganisms (PPM) *H. influenzae*, *M. catarrhalis* and *S. pneumoniae*. PPM positive was defined as total load ≥1 × 10^4^copies/ml. Sputum and whole blood were analysed for differential cell counts.

**Results:**

At baseline, bacterial counts were not related to blood eosinophils, but sputum eosinophil % was significantly lower in patients with PPM positive compared to PPM negative samples (medians: 0.5% vs. 1.25% respectively, *p* = 0.01). Patients with PPM positive samples during an exacerbation had significantly lower blood eosinophil counts at exacerbation compared to baseline (medians: 0.17 × 10^9^/L vs. 0.23 × 10^9^/L respectively, *p* = 0.008), while no blood eosinophil change was observed with PPM negative samples.

**Conclusions:**

These findings indicate an inverse relationship between bacterial infection and eosinophil counts. Bacterial infection may influence corticosteroid responsiveness by altering the profile of neutrophilic and eosinophilic inflammation.

**Electronic supplementary material:**

The online version of this article (doi:10.1186/s12931-017-0570-5) contains supplementary material, which is available to authorized users.

## Background

COPD is a heterogeneous condition, composed of different clinical and pathophysiological components that vary both in presence and severity between patients [1]. This heterogeneity causes variability in the responses to pharmacological treatments. Biomarkers that predict treatment responses to anti-inflammatory drugs may be useful for optimising the benefit versus risk ratio.

Sputum eosinophil counts in stable COPD patients predict the clinical response to corticosteroids [2, 3]. However, measuring sputum eosinophils is time consuming, and some patients do not provide adequate samples for analysis. Blood eosinophil measurements are more practical, and appear to be a surrogate biomarker for sputum eosinophils as these measurements show a degree of correlation within the same individual, both in stable COPD patients and during exacerbations [4–7]. Retrospective analysis of COPD clinical trials have shown that higher blood eosinophil counts predict a greater reduction in exacerbation rates with inhaled corticosteroid/long acting beta agonist (ICS/LABA) combinations compared to LABA [8–10]. Furthermore, oral corticosteroid treatment during exacerbations has a greater effect in COPD patients with higher blood eosinophil counts [11].

Chronic bacterial infection in COPD patients causes greater neutrophilic inflammation in the lungs [12–14]. Neutrophilic lung inflammation responds poorly to corticosteroids [15–17], implicating bacterial infection as a potential cause of corticosteroid insensitivity in COPD patients. The relationship between bacterial infection and eosinophil counts is not established in COPD. There may be an inverse relationship between these parameters, as blood eosinophil counts are known to be reduced during severe bacterial infection [18, 19]. The existence of such an inverse relationship would suggest that the interaction between bacterial infection and eosinophils determines the corticosteroid response in COPD patients.

The primary aim of the analysis reported here using data from the COPDMAP cohort was to test the hypothesis that the bacterial load and eosinophil counts are inversely related in COPD patients. We investigated the relationship between eosinophil counts (in the blood and sputum) and the bacterial load in the stable state and during exacerbations in COPD patients.

## Methods

### Subjects

COPD patients aged ≥ 40 years were recruited at 3 sites (Manchester, Leicester and London) and enrolled into the COPDMAP prospective observational cohort study (www.copdmap.org). Patients had a physician diagnosis of COPD, post-bronchodilator forced expiratory volume in 1 s (FEV_1_)/forced vital capacity (FVC) ratio <0.7, ≥10 pack year smoking history and no previous asthma diagnosis. The patients included in this analysis were those who provided a blood sample for eosinophil analysis and a sputum sample for bacterial quantification at the baseline visit. All patients provided written informed consent using protocols approved by the local Ethics Committees (11/L0/1630; 10/H/1003/108; 07/H0406/157).

Additional methods are in the online supplement (Additional file 1).

### Study design

Patients were seen at baseline and at exacerbation onset. Symptoms were assessed using the modified MRC Scale (mMRC) and the COPD assessment test (CAT). Health related quality of life using the St George’s Respiratory Questionnaire (SGRQ-C) and fat free mass index (FFMI) were assessed. Lung function measurements and 6 min walk test (6MWT) were performed according to guidelines [20, 21]. Spontaneous and/or induced sputum was obtained as previously described [22] and blood samples sent to local hospital laboratories.

Patients contacted the research team if they experienced a change in symptoms consistent with an acute exacerbation. Daily diary cards were used. Patients were assessed by a clinician and exacerbations defined as in increase in two respiratory symptoms (with at least one major symptom) for two consecutive days [23]. Blood and sputum sampling were performed prior to any systemic therapy including treatment with oral corticosteroids and/or antibiotics. Samples from the first exacerbation event per patient were used in this analysis.

### Sputum and blood analysis

All patients attempted to produce a spontaneous sample at baseline and exacerbation. A sputum induction was attempted (safety permitting) if an insufficient spontaneous sample was produced. Spontaneous or induced sputum was processed for quantitative polymerase chain reaction (qPCR) detection of the common respiratory potentially pathogenic microorganisms (PPM) *H. influenzae*, *M.catarrhalis* and *S. pneumoniae* and for human rhinovirus (RV) as previously described [24, 25]. Patients were categorised as PPM positive if the total load was above 1 × 10^4^copies/ml or RV positive if the load was greater than 1 × 10^1^copies/ml [26, 27]. Sputum was also processed for differential cell counts (Additional file 2: Figure S1) [28]. However, small sputum samples (minimum weight of approximately 0.075 g) were preferentially processed for qPCR analysis only. Whole blood was analysed for differential leukocyte count and the blood eosinophil count measured at baseline and exacerbation were used in this analysis.

### Statistical analysis

Statistical analysis for non-parametric data was performed using; Wilcoxon signed rank test for within individual comparison or the Mann–Whitney *U* test for between group comparison and chi-square test for categorical variables. Receiver operating characteristics (ROC) curves determined if blood eosinophil counts predict a sputum eosinophil count of ≥3%. Spearman’s correlation test assessed associations between variables. *P* < 0.05 was considered statistically significant. Analyses were performed using GraphPad Prism version 6.00 (San Diego, USA).

## Results

### Stable state microbiology and eosinophils

#### Blood eosinophil counts

One hundred sixty-eight patients had a blood eosinophil and sputum qPCR bacterial measurement at baseline. The demography of this population is shown in Table 1; the mean post-bronchodilator FEV_1_ was approximately 57% predicted, with a median exacerbation rate in the previous year of 1, and mean CAT and SGRQ scores of 18.7 and 47.8 respectively. 52% of patients had PPM positive samples (defined by a threshold value of ≥1 × 10^4^). There were no differences in the blood eosinophil count (medians: 0.21 × 10^9^/L vs. 0,18 × 10^9^/L, *p* = 0.50), sputum cells counts (all *p* > 0.05) and total bacterial load (medians: 3.55 × 10^4^ vs.5.38 × 10^4,^ *p* = 0.52) between ICS users and non-users respectively.

**Table 1:** Baseline demographics of the patients enrolled onto the study

Characteristics	n	
Age, yrs	168	69.8 (8.1)
Gender, (% Male)	168	74
Current Smoker, (%)	168	32
Inhaled steroid use (%)	168	86
Inhaled steroid dose (μg) (BDP equivalent)	168	1000 [200–2000]
LABA use (%)	168	88
LAMA use (%)	168	78
Pack Years	168	47.0 [10.0–220.0]
BMI (kg/m^2^)	168	26.2 [18.0–45.0]
FFMI (kg/m^2^)	159	17.8 (3.5)
Chronic Bronchitis (%)	164	73
Exacerbation rate (prior study entry)	168	1.0 [0.0–15.0]
SGRQ total	162	47.8 (18.4)
mMRC score	145	2.0 [1.0–4.0]
CAT score	165	18.7 (7.1)
6MWD	132	380.9 (109.4)
KCO, %	119	75.4 (27.0)
Pre FEV_1_ (L)	104	1.5 (0.6
Pre FEV_1_%	104	55.7 (19.9)
Pre FVC (L)	104	3.0 (0.9)
Pre FEV_1_/FVC	104	0.5 (0.1)
Post FEV_1_ (L)	168	1.5 (0.6)
Post FEV_1_%	168	56.8 (18.9)
Post FVC (L)	168	3.0 (0.9)
Post FEV_1_/FVC	168	0.5 (0.1)
Reversibility %	105	7.2 [−13.0–32.5]
Reversibility ml	105	100 [−180–420]
Sputum Total Cell Count x10^6^/g	90	3.7 [0.2–84.6]
Sputum Neutrophil %	94	76.0 [1.0–99.3]
Sputum Eosinophil %	94	0.8 [0.0–13.2]
Sputum Neutrophil cell count x10^6^/g	90	2.5 [0.0–82.4]
Sputum Eosinophil cell count x10^6^/g	90	0.03 [0.0–0.5]
PPM positive (≥1 × 10^4^ copies/ml), %	168	52

Summaries are presented as mean (SD), median [Range] and percentages as appropriate

Definitions of abbreviations: *BDP* Beclometasone dipropionate equivalent, *LABA* Long Acting Beta Agonist, *LAMA* Long Acting muscarinic antagonist, *BMI* Body Mass Index, *FFMI*, Fat Free Mass Index; *SGRQ* St George’s Respiratory Questionnaire, *mMRC* modified Medical Research Council, *CAT* COPD Assessment Test, *6MWD* 6 min walk test, *FEV*_*1*_ Forced Expired Volume in first second, *FVC* Forced vital capacity, *PPM* Potentially pathogenic microorganism, *Pre* Pre bronchodilator, *Post* Post bronchodilator

**Fig. 1:**
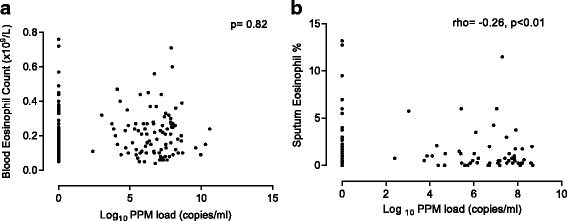
The correlation between bacterial load and **a** blood eosinophil count and **b** sputum eosinophil % at baseline

**Fig. 2:**
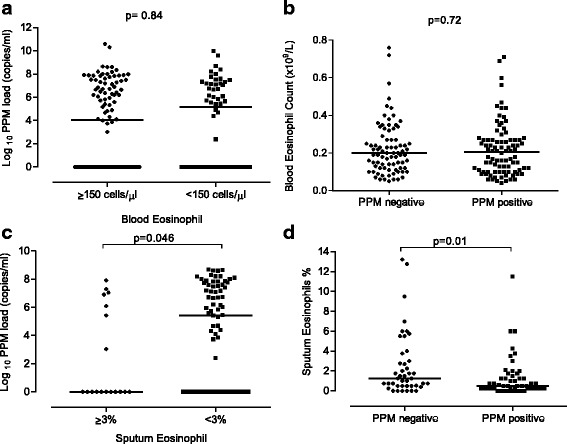
The relationship between eosinophils and bacterial load at baseline; **a** the bacterial load in patients with blood eosinophil ≥ 150 cells/μl and < 150 cells/μl, **b** the blood eosinophil count between PPM negative and PPM positive groups, **c** the bacterial load in patients with sputum eosinophil count ≥ 3% and < 3%, **d** the sputum eosinophil % between PPM negative and PPM positive groups

There were no significant associations between the blood eosinophil count and bacterial load (Fig. 1a), and no difference in bacterial load using different blood eosinophil threshold values i.e. ≥ and <150 cells/μl (Fig. 2a), 2% or 300 cell/μl (not shown). Additionally, there were no differences in blood eosinophil counts between patients with PPM positive and PPM negative samples (Fig. 2b).

#### Sputum eosinophil counts

Ninety-four of the 168 patients produced a sufficient sputum sample for differential cell count analysis; of which 75 and 19 were from spontaneous and induced samples respectively. Total cell count (x10^6^/g) in the spontaneous samples were lower compared to induced samples (medians: 2.75 vs. 6.59 respectively, *p* = 0.003), but no differences were observed for sputum % differential counts including sputum eosinophil % (medians 0.75 vs. 0.6 respectively, *p* = 0.82).

Figure 2c shows that patients with a sputum eosinophil count ≥3% had a significantly lower bacterial load compared to those with a sputum eosinophil <3% (*p* = 0.046). The inflammatory cell profiles of the patients with sputum eosinophil count <3% and ≥3% are presented in Table 2; there was a higher neutrophil percentage (medians 80.1% vs. 68.4% respectively) and lower absolute eosinophil cell count (medians 0.2 × 10^6^/g versus 0.0 × 10^6^/g respectively) in the former group.

**Table 2:** PPM load and sputum inflammatory cell counts in patients with and without eosinophilic inflammation

Sputum	≥3% (*n* = 18)	<3% (*n* = 76)	*p* value
PPM load (copies/ml)	0.0 [0.0–8.2 × 10^7^]	2.7 × 10^5^ [0–4.9 × 10^8^]	*0.046*
Neutrophil %	68.4 [7.0–89.8]	80.1 [1.0–99.3]	*0.04*
Macrophage%	17.4 [2.9–85.5]	15.3 [0.5–88.5]	0.27
Eosinophil %	5.9 [3.0–13.2]	0.5 [0.0–3.0]	*<0.0001*
Lymphocyte %	0.0 [0.0–3.0]	0.0 [0.0–6.0]	0.16
Epithelial %	4.0 [0.5–33.0]	2.5 [0.0–89.3]	0.26
Total cell count x10^6^/g	4.0 [0.2–9.9]	3.4 [0.2–84.6]	0.62
Neutrophil cell count x10^6^/g	2.1 [0.0–7.7]	2.5 (0.0–82.4]	0.38
Macrophage cell count x10^6^/g	0.7 [0.0–2.3]	0.5 [0.1–4.2]	0.68
Eosinophil cell count x10^6^/g	0.2 [0.0–0.5]	0.0 [0.0–0.4]	*<0.0001*
Lymphocyte cell count x10^6^/g	0.0 [0.0–0.1]	0.0 [0.0–0.3]	0.13
Epithelial cell count x10^6^/g	0.1 [0.0–0.6]	0.10 [0.00–2.3]	0.46

Summaries are presented as median [Range]

**Fig. 3:**
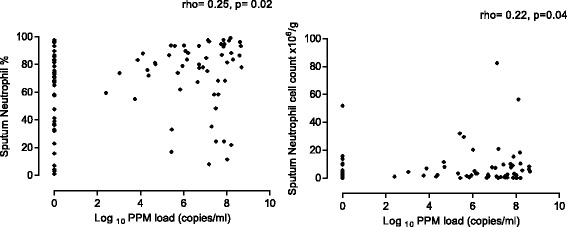
The correlation between bacterial load and both sputum neutrophil % and absolute count at baseline

The sputum eosinophil % was significantly lower in patients with PPM positive samples compared to PPM negative samples (medians: 0.5% vs. 1.25%, *p* = 0.01, Fig. 2d). Sputum eosinophil % was inversely correlated with bacterial load (rho = −0.26, *p* < 0.01, Fig. 1b). This association was not evident with the sputum eosinophil absolute count (*p* = 0.5). There was a significant positive correlation between the bacterial load and both sputum neutrophil % and absolute cell count (rho = 0.25, *p* = 0.02 and rho = 0.22, *p* = 0.04 respectively, Fig. 3).

### Exacerbation microbiology and eosinophils

There were 109 exacerbation events that had blood eosinophil counts at both baseline and exacerbation, and qPCR bacterial counts at exacerbation. The exacerbations occurred a mean 171 days after the baseline visit; 78 exacerbations were PPM positive and 31 were PPM negative. A subset of 70 patients also had a baseline qPCR bacterial count. Sputum eosinophil % counts at baseline and exacerbation were available in 30 of these patients; 22 were PPM positive and 8 were PPM negative (Additional file 3: Figure S2 shows a summary of the number of patients with blood/sputum eosinophil counts and qPCR data at exacerbation alone, or coupled to baseline measurements).

The differences in blood counts, sputum counts and bacterial load between baseline and exacerbation in the whole population are presented in Table 3. White blood cell count (WBC), blood neutrophil and monocyte counts were significantly increased at exacerbation, while there was no difference for the blood eosinophil count. Bacterial load, total sputum cell count (x10^6^/g) and the absolute and % sputum neutrophil count were higher at exacerbation. The absolute and % sputum macrophage counts and sputum eosinophil % were significantly lower at exacerbation.

**Table 3:** Comparison of blood, sputum and bacterial counts between baseline and exacerbation

	n	Baseline	Exacerbation	*p* value
Total white blood cell count (x10^9^/L)	109	7.35 [4.40–15.60]	8.90 [2.80–20.50]	<0.0001
Neutrophil blood count (x10^9^/L)	109	4.69 [2.55–12.23]	6.01 [1.42–18.12]	<0.0001
Eosinophil blood count (x10^9^/L)	109	0.23 [0.04–0.90]	0.18 [0.00–0.78]	0.22
Lymphocyte blood count (x10^9^/L)	109	1.70 [0.75–4.25]	1.80 [0.44–4.66]	0.133
Monocyte blood count (x10^9^/L)	109	0.57 [0.28–1.41]	0.70 [0.26–1.86]	<0.0001
Total Cell count (x10^6^/g)	30	4.07 [0.25–84.56]	8.67 [0.41–154.5]	0.02
Sputum Neutrophil cell count (x10^6^/g)	30	3.34 [0.02–82.44]	7.20 [0.08–151.6]	0.03
Sputum Macrophage cell count (x10^6^/g)	30	0.74 [0.04–4.22]	0.09 [0.00–1.55]	<0.0001
Sputum Eosinophil cell count (x10^6^/g)	30	0.06 [0.00–0.52]	0.01 [0.00–3.20]	0.11
Sputum Lymphocyte cell count (x10^6^/g)	30	0.00 [0.00–0.25]	0.00 [0.00–0.09]	0.46
Sputum Epithelial cell count (x10^6^/g)	30	0.19 [0.00–2.32]	0.17 [0.00–0.95]	0.39
Sputum Neutrophil %	30	77.13 [3.00–97.75]	89.00 [5.25–99.25]	0.04
Sputum Macrophage %	30	15.88 [1.50–88.50]	6.75 [0.50–70.25]	0.02
Sputum Eosinophil %	30	0.92 [0.00–12.75]	0.38 [0.00–86.5]	0.03
Sputum Lymphocyte %	30	0.13 [0.00–2.50]	0.00 [0.00–1.00]	0.06
Sputum Epithelial cell %	30	3.94 [0.00–40.00]	2.89 [0.00–54.76]	0.74
Total Bacterial Load (genome copies/ml)	70	2.85 × 10^3^ [0.0–3.69 × 10^10^]	5.13 × 10^5^ [0.0–6.09 × 10^8^]	0.02

#### Blood eosinophil counts

**Fig. 4:**
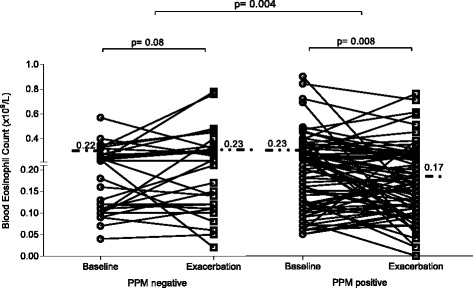
The blood eosinophil count at baseline and exacerbation in patients defined as PPM positive or PPM negative at exacerbation (PPM threshold value of 1 × 10^4^). PPM = potentially pathogenic microorganisms. Dotted lines represent median values

Table 4 presents a comparison of the blood counts between baseline and exacerbation for the PPM positive and negative groups. Patients with PPM positive samples (using a threshold value of ≥1 × 10^4^) during an exacerbation had significantly lower blood eosinophil counts at exacerbation compared to baseline (medians: 0.17 × 10^9^/L vs. 0.23 × 10^9^/L respectively, *p* = 0.008, Fig. 4). There was no change in the blood eosinophil count at exacerbation compared to baseline in patients with PPM negative samples at exacerbation (medians: 0.23 × 10^9^/L vs. 0.22 × 10^9^/L respectively). The median decrease in blood eosinophil count between the baseline and exacerbation visit was significantly different in PPM positive compared to PPM negative patients (medians: −0.02 × 10^9^/L vs. 0.04 × 10^9^/L respectively, *p* = 0.004, Fig. 4). A similar pattern was observed using a PPM threshold of ≥1 × 10^6^ at exacerbation (Additional file 4: Figure S3).

**Table 4:** Comparison of blood and sputum counts between baseline and exacerbation for PPM positive and PPM negative groups

BLOOD	PPM positive *n* = 78			PPM negative *n* = 31		
	Baseline	Exacerbation	*p* value	Baseline	Exacerbation	*p* value
White blood cell count (x10^9^/L)	7.4 [4.4–15.6]	8.9 [2.8–20.5]	<0.0001	7.00 [5.03–13.4]	8.90 [4.90–15.40]	<0.0001
Neutrophil count (x10^9^/L)	4.95 [2.95–12.23]	6.13 [1.42–18.12]	<0.0001	4.38 [2.97–8.29]	5.50 [2.36–10.98]	0.004
Eosinophil blood count (x10^9^/L)	0.23 [0.05–0.90]	0.17 [0.00–0.76]	0.008	0.22 [0.04–0.57]	0.23 [0.02–0.78]	0.08
Lymphocyte blood count (x10^9^/L)	1.70 [0.75–3.67]	1.89 [0.44–4.66]	0.13	1.67 [1.00–4.25]	1.79 [0.50–3.03]	0.69
Monocyte blood count (x10^9^/L)	0.55 [0.28–1.41]	0.68 [0.26–1.87]	<0.0001	0.6 [0.4–1.06]	0.77 [0.42–1.43]	<0.0001
SPUTUM	PPM positive *n* = 22			PPM negative *n* = 8		
	Baseline	Exacerbation	*p* value	Baseline	Exacerbation	*p* value
Total Cell count (x10^6^/g)	5.84 [0.63–84.56]	10.58 [0.41–154.5]	0.14	3.01 [0.25–5.27]	6.51 [0.60–36.67]	0.008
Sputum Neutrophil count (x10^6^/g)	5.14 [0.02–82.44]	9.79 [0.08–151.6]	0.11	0.73 [0.16–3.61]	5.19 [0.19–35.75]	0.05
Sputum Macrophage count (x10^6^/g)	0.74 [0.14–4.22]	0.11 [0.00–1.55]	<0.0001	0.74 [0.04–2.16]	0.07 [0.01–0.37]	0.02
Sputum Eosinophil count (x10^6^/g)	0.07 [0.00–0.52]	0.01 [0.00–0.31]	0.09	0.02 [0.01–0.50]	0.03 [0.00–3.17]	0.61
Sputum Lymphocyte count (x10^6^/g)	0.00 [0.00–0.25]	0.00 [0.00–0.27]	0.11	0.00 [0.00–0.01]	0.005 [0.00–0.09]	0.31
Sputum Epithelial count (x10^6^/g)	0.19 [0.00–2.32]	0.16 [0.00–0.84]	0.56	0.20 [0.04–0.30]	0.24 [0.03–0.95]	0.74
Sputum Neutrophil %	84.00 [3.00–97.75]	90.75 [19.00–99.25]	0.21	55.38 [7.00–83.53]	78.00 [5.25–97.5]	0.11
Sputum Macrophage %	11.12 [1.50–88.50]	6.00 [0.50–70.25]	0.28	26.88 [8.82–85.5]	9.00 [1.5–35.5]	0.02
Sputum Eosinophil %	0.75 [0.00–6.98]	0.23 [0.00–2.00]	0.046	1.88 [0.29–12.75]	1.00 [0.00–86.5]	0.38
Sputum Lymphocyte %	0.00 [0.00–0.25]	0.00 [0.00–1.00]	0.35	0.00 [0.00–2.50]	0.13 [0.00–0.25]	0.63
Sputum Epithelial cell %	2.13 [0.00–40.00]	2.13 [0.00–54.76]	0.89	7.68 [1.50–17.50]	5.00 [0.75–11.00]	0.25

The proportion of individuals with a decrease in blood eosinophil counts at exacerbation from baseline was significantly higher in PPM positive patients compared to PPM negative patients (53% vs 29%, *p* = 0.02). 4/78 (5%) of the PPM positive and 1/31 (3%) of the PPM negative patients had blood eosinopenia (counts ≤0.04 × 10^9^/L).

A significant increase in the blood neutrophil count was observed at exacerbation compared to baseline in patients with PPM positive (medians: 6.13 × 10^9^/L vs. 4.95 × 10^9^/L respectively, *p* < 0.0001) and negative samples (5.50 × 10^9^/L vs 4.38 × 10^9^/L respectively, *p* = 0.004) during exacerbation (Table 4). There was no significant difference in the median change between the 2 groups (*p* > 0.99). In addition, the total WBC and blood monocyte count were significantly increased at exacerbation compared to baseline in both the PPM positive and negative groups (Table 4).

Seventy-five of the 109 patients had rhinovirus load measured at exacerbation; 16 were rhinovirus positive and 59 were rhinovirus negative. 75% and 71% of the rhinovirus positive and negative samples were PPM positive respectively. There were no changes in blood eosinophil counts between the baseline and exacerbation visit in the rhinovirus positive (medians: 0.26 vs. 0.20 respectively, *p* = 0.87) and negative (medians: 0.22 vs. 0.17 respectively, *p* = 0.34) groups (Additional file 5: Table S1).

#### Sputum eosinophil counts

**Fig. 5:**
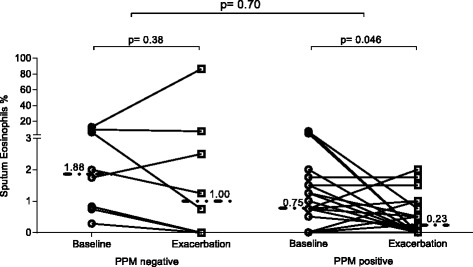
The sputum eosinophil count at baseline and exacerbation in patients defined as PPM positive or PPM negative at exacerbation (PPM threshold value of 1 × 10^4^). PPM = potentially pathogenic microorganisms. Dotted lines represent median values

Sputum eosinophil % decreased significantly at exacerbation compared to baseline in patients who were PPM positive (medians: 0.23% vs. 0.75% respectively, *p* = 0.046 & Fig. 5). No significant change was observed in patients who were PPM negative at exacerbation (Fig. 5). The comparison of the eosinophil count change from baseline to exacerbation revealed no difference between PPM positive and PPM negative patients. When repeating the analysis using sputum neutrophil % in both the PPM positive and negative groups, we observed a numerical but non-significant increase in both groups (Additional file 6: Figure S4). Comparisons of the other sputum cell counts are presented further in Table 4.

### Relationship between blood and sputum eosinophils

**Fig. 6:**
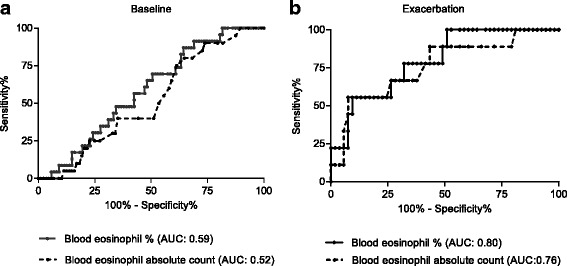
ROC curves of blood eosinophil counts as a predictor of sputum eosinophilia at **a** baseline and **b** exacerbation

One hundred ten patients had both a blood and sputum eosinophil count available at baseline, while 62 patients had a blood and sputum eosinophil count available during their first exacerbation event. The baseline blood eosinophil % and absolute counts were weakly correlated with sputum eosinophil % (rho = 0.24, *p* = 0.01 and rho = 0.19, *p* = 0.05 respectively) and baseline blood eosinophil % and absolute counts did not predict sputum eosinophil % (area under the ROC curve (AUC) 0.59, *p* = 0.21 and AUC 0.52, *p* = 0.79 respectively, Fig. 6a). In contrast, at exacerbation there was a stronger correlation for blood eosinophil % (rho = 0.41, *p* = 0.001) and absolute counts (rho = 0.43, *p* < 0.001) with sputum eosinophil %. Both blood eosinophil % and absolute count were good predictors of sputum eosinophil % at exacerbation (AUC 0.80, *p* = 0.005 and AUC 0.76, *p* = 0.01 respectively, Fig. 6b).

## Discussion

We observed evidence of an inverse relationship between bacterial infection and eosinophil counts in COPD. Firstly, sputum eosinophil counts were lower in COPD patients with bacterial infection in the stable state. Secondly, COPD patients with bacterial infection during exacerbations had a significant decrease in the blood eosinophil absolute count compared to the stable state, while no blood eosinophil count changes were observed in patients without bacterial infection. These observations during the stable state and exacerbations showing an inverse relationship between bacterial infection and eosinophil counts may be relevant to corticosteroid responsiveness in COPD; the increased corticosteroid responsiveness observed with higher eosinophil counts could be due, at least partly, to lower levels of bacterial infection..

This inverse relationship between bacterial counts and blood eosinophils was present during exacerbations but not in the stable state. This is likely to be due to the stronger association between blood and sputum eosinophils at exacerbation compared to the stable state. Blood eosinophils have been proposed as a surrogate biomarker of sputum eosinophils, but the weak relationship between these measurements in the stable state, demonstrated here and in previous studies [6, 7], highlights a limitation of blood eosinophil counts in this context.

The prevalence of bacterial colonisation at baseline in our study (52%) was comparable to previously published studies that used qPCR to detect PPM presence; Bafadhel et al., [12] reported a 51% prevalence while, Barker et al., [26] reported a 77% prevalence. In the stable state, higher bacterial counts were associated with higher sputum neutrophil % and lower eosinophil %. This relationship between higher sputum bacterial counts and increased sputum neutrophil % is known [12, 13, 29]. The lower sputum eosinophil % in COPD patients with bacterial colonisation is partly due to the bacterial driven increase in absolute and percentage neutrophil counts causing a lower calculated eosinophil %. However, the absolute sputum eosinophil count is not dependent on any calculation involving the neutrophil count, and we observed that the absolute sputum eosinophil count was also lower in patients with sputum eosinophils <3%, indicating a genuine reduction in eosinophil numbers in the airways of patients with higher bacterial counts.

The inverse relationships between bacterial counts and sputum eosinophils provide a potential mechanism that determines corticosteroid responsiveness in COPD patients. Individuals with lower sputum eosinophil counts have higher levels of bacterial infection; perhaps the presence of more bacteria, with associated neutrophilic inflammation [12–14], contributes to the lower corticosteroid response previously reported [16, 17]. Neutrophilic airway inflammation appears to be poorly responsive to corticosteroid treatment [15, 17, 30], and the absence of bacterial inflammation may therefore favour a greater corticosteroid response through a shift in the balance of airway inflammation away from neutrophilic inflammation towards more eosinophilic inflammation.

Previous studies have failed to show a significant difference in sputum eosinophil % between stable COPD patients with bacteria present versus those without, although a numerical decrease in the former group was observed [12, 26]. Compatible with our results is the previous finding of a significantly lower level of sputum CCL13 (an eosinophil associated cytokine) in PPM positive COPD patients [26].

It has been reported that the effects of oral corticosteroids are dependent on the blood eosinophil count during COPD exacerbations, with more likelihood of treatment failure or a longer hospital stay with lower blood eosinophil counts [11, 31, 32]. The data reported here show lower eosinophil counts during acute bacterial infections, and it could be inferred that treatment failure with oral corticosteroids in such cases is related to the presence of airway bacteria. During exacerbations both sputum and blood eosinophil measurements showed similar relationships to bacterial counts. However, the sputum data were less robust due to smaller sample size.

Blood neutrophil counts are known to increase during infection, irrespective of bacterial presence [33]; we observed the same during COPD exacerbations. The decrease in blood eosinophil absolute count during exacerbation cannot be due to the increase in blood neutrophil counts for two reasons. First, there was a similar increase in blood neutrophil counts in both PPM positive and negative patients; second, we measured blood eosinophil absolute numbers which are not influenced by calculations of percentage relative to neutrophils.

In COPD patients hospitalised for exacerbations, blood eosinopenia is an independent predictor of mortality and length of stay [34, 35]. The exacerbations in this study were treated in the outpatient setting (only 3/109 exacerbations resulted in hospitalisation). These less severe events had few cases of eosinopenia (5/109 patients). Nevertheless, our results demonstrate an effect of airway bacteria on blood eosinophil counts even in patients without overt bacterial sepsis. Similarly, blood eosinophil counts decrease in asthma patients with bacterial infections [36].

Rhinovirus infection had no effect on blood eosinophil counts. Experimental rhinovirus infection causes an increase in the airway bacterial load, demonstrating the complexity of the relationship between virus and bacterial infection during COPD exacerbations [37]. Our results indicate that bacterial, rather than viral, infection modulates blood eosinophil counts. However, it is worth noting that due to insufficient sputum samples being available, rhinovirus detection was performed in a smaller proportion of patients (75/109 patients). Consequently, the lack of effect of rhinovirus on eosinophil counts could potentially be attributed to limited power in the analysis.

The mechanism responsible for the decrease in eosinophil counts during bacterial infection is unclear. Eosinophils can trigger innate immune responses to pathogens through the release of extracellular DNA traps and the expression of specific pattern recognition receptors including Toll like receptor 4 [38, 39]. Furthermore, eosinophil granule proteins have bactericidal activity [40]. A decrease in circulating eosinophils may be a result of adrenal glucocorticoid stimulation in response to the stress of bacterial infection or the rapid accumulation of eosinophils at the inflammatory site [41, 42].

A limitation of this study is that we defined a bacteria positive sample based on the total load of bacteria measured rather than a species specific count. This was done to increase statistical power to address the relationship between eosinophils and the common pathogenic bacteria in COPD. However, it is important to note that the total bacterial load from each bacteria positive sample had at least one of the three quantified pathogens above the 1 × 10^4^ threshold used. The presence of other important bacteria, such as *Pseudomonas aeruginosa,* was not assessed using qPCR. Additionally, we cannot discount the possibility of selection bias as a proportion of our patients failed to provide an adequate, high quality sputum sample for differential cell count analysis. The majority of the sputum samples used in our study were spontaneously produced and although cell viability can be lower in spontaneous samples [43], we observed no differences in % sputum counts between spontaneous and induced samples, including the sputum eosinophil % count as previously reported [43]. Finally, the majority of our patients were using ICS, but we found no difference in sputum or blood cell counts or bacterial loads due to ICS use. This is in keeping with studies that have shown no effect of ICS on blood or sputum eosinophil counts [2, 44].

Future studies of the effects of ICS during the stable state could measure sputum (and blood) eosinophil counts and bacterial loads to test the hypothesis that increased bacterial load associated with reduced sputum eosinophil counts predicts reduced therapeutic response. Similar studies using oral corticosteroids during acute exacerbations measuring blood eosinophil counts and bacterial loads could test the hypothesis that increased bacterial load associated with reduced blood eosinophil counts predicts reduced therapeutic response.

## Conclusion

In conclusion, our results show an inverse relationship between sputum eosinophils and airway bacterial load during the stable state, while a decrease in blood eosinophil counts occurs in COPD exacerbations with bacterial presence. These inverse relationships between eosinophil measurements and bacterial counts are potentially important determinants of individual responses to corticosteroid treatment.

## Additional file

Additional file 1: Additional methods supplement. (DOCX 38.7 kb)
Additional file 2: Figure S1. Outline of the methods used for the processing of sputum qPCR and differential cell count samples. (PPTX 64.1 kb)
Additional file 3: Figure S2. A summary of the number of patients with blood / sputum eosinophil counts and qPCR data at exacerbation alone, or coupled to baseline measurements. PPM=potentially pathogenic microorganisms. (PPTX 51.6 kb)
Additional file 4: Figure S3. The blood eosinophil count at baseline and exacerbation in patients defined as PPM positive or PPM negative at exacerbation (PPM threshold value of 1x10 ). PPM=potentially pathogenic microorganisms. Dotted lines represent median values. (PPTX 274 kb)
Additional file 5: Table S1. The change in blood eosinophil count between RV negative and RV positive exacerbations. (PPTX 18.8 kb)
Additional file 6: Figure S4. The sputum neutrophil % at baseline and exacerbation in patients defined as PPM positive or PPM negative at exacerbation. PPM=potentially pathogenic microorganisms. Dotted lines represent median values. (PPTX 189 kb)

## Abbreviations

COPD: Chronic obstructive pulmonary disease
GOLD: Global initiative for chronic obstructive
ICS: Inhaled corticosteroid
LABA: Long acting beta agonist
FEV_1_: Forced expiratory volume in 1 s
FVC: Forced vital capacity
mMRC: modified MRC scale
CAT: COPD assessment test
SGRQ-C: St George’s respiratory questionnaire
FFMI: Fat free mass index
6MWT: 6 min walk test
qPCR: quantitative polymerase chain reaction
PPM: Potentially pathogenic microorganisms
RV: Rhinovirus
ROC: Receiver operating curves
AUC: Area under the curve
