# Supplementary material for: Blood and sputum eosinophils in COPD; relationship with bacterial load
Source: Respir Res. 2017 May 8;18:88. doi: 10.1186/s12931-017-0570-5 (PMC5422866; doi:10.1186/s12931-017-0570-5)
Supplement: Additional file 1: — Additional methods supplement. (DOCX 38.7 kb) [file 12931_2017_570_MOESM1_ESM.docx]

**Methods**

***Subjects***

All patients had a physician diagnosis of COPD, post-bronchodilator forced expiratory volume in 1 second (FEV_1_) forced vital capacity (FVC) ratio <0.7, ≥10 pack year smoking history and no previous asthma diagnosis. Patients with significant bronchodilator reversibility or history of atopy were not excluded. Patients on maintenance oral corticosteroids or azithromycin were excluded from this analysis.

***Stable Measurements***

Stable state was defined as no symptom-defined exacerbations or systemic treatment with antibiotics and/or steroids in the preceding four weeks***.*** Symptoms and health related quality of life were assessed using the modified MRC Scale ([E1](#_ENREF_1)), the COPD assessment test (CAT) ([E2](#_ENREF_2)) and the St George’s Respiratory Questionnaire (SGRQ-C) ([E3](#_ENREF_3)). Lung function measurements (spirometry, plethysmography and gas transfer) and functional capacity using the 6 minute walk test (6MWT) were performed in accordance with European Respiratory Society (ERS)/American Thoracic Society (ATS) recommendations ([E4](#_ENREF_4), [E5](#_ENREF_5), [E6](#_ENREF_6), [E7](#_ENREF_7)). Reversibility was performed using salbutamol 400mcg. Fat free mass index (FFMI) was determined by bioelectrical impedance.

***Exacerbation measurements***

Patients contacted the research team if they experienced a change in symptoms consistent with an acute exacerbation. Daily diary cards were used. Patients were assessed by a clinician and exacerbations were defined as increase in two respiratory symptoms (with at least one major symptom) for two consecutive days ([E8](#_ENREF_8)).

***Sputum and Blood Analysis***

Spontaneous or induced sputum was processed for quantitative polymerase chain reaction (qPCR) detection of the common respiratory potentially pathogenic microorganisms (PPM) *H. influenzae*, *M.catarrhalis* and *S. pneumoniae* and for human rhinovirus (RV) as previously described ([E9](#_ENREF_9), [E10](#_ENREF_10)). Briefly, selected sputum plugs was weighed and suspended in 8 times volume of phosphate-buffered saline (PBS). Glass beads were added to this suspension and it underwent homogenisation by vortexing for 15s, rocking for 15 min and vortexing for an additional 15s. Aliquots of 500μl were stored at -80^o^C for subsequent qPCR processing. Patients were categorised as PPM positive if the total load was above 1x104copies/ml or RV positive if the load was greater than 1x101copies/ml ([E11](#_ENREF_11), [E12](#_ENREF_12)).

**REFERENCES**

E1. Mahler DA, Wells CK. Evaluation of clinical methods for rating dyspnea. Chest. [Comparative Study

Research Support, Non-U.S. Gov't

Research Support, U.S. Gov't, P.H.S.]. 1988 Mar;93(3):580-6.

E2. Jones PW, Harding G, Berry P, Wiklund I, Chen WH, Kline Leidy N. Development and first validation of the COPD Assessment Test. Eur Respir J. 2009 Sep;34(3):648-54.

E3. Meguro M, Barley EA, Spencer S, Jones PW. Development and Validation of an Improved, COPD-Specific Version of the St. George Respiratory Questionnaire. Chest. 2007 Aug;132(2):456-63.

E4. ATS statement: guidelines for the six-minute walk test. American journal of respiratory and critical care medicine. 2002 Jul 1;166(1):111-7.

E5. Miller MR, Hankinson J, Brusasco V, Burgos F, Casaburi R, Coates A, et al. Standardisation of spirometry. Eur Respir J. 2005 Aug;26(2):319-38.

E6. Wanger J, Clausen JL, Coates A, Pedersen OF, Brusasco V, Burgos F, et al. Standardisation of the measurement of lung volumes. The European respiratory journal. [Review]. 2005 Sep;26(3):511-22.

E7. Macintyre N, Crapo RO, Viegi G, Johnson DC, van der Grinten CP, Brusasco V, et al. Standardisation of the single-breath determination of carbon monoxide uptake in the lung. The European respiratory journal. 2005 Oct;26(4):720-35.

E8. Anthonisen NR, Manfreda J, Warren CP, Hershfield ES, Harding GK, Nelson NA. Antibiotic therapy in exacerbations of chronic obstructive pulmonary disease. Ann Intern Med. 1987 Feb;106(2):196-204.

E9. Garcha DS, Thurston SJ, Patel AR, Mackay AJ, Goldring JJ, Donaldson GC, et al. Changes in prevalence and load of airway bacteria using quantitative PCR in stable and exacerbated COPD. Thorax. [Research Support, Non-U.S. Gov't]. 2012 Dec;67(12):1075-80.

E10. George SN, Garcha DS, Mackay AJ, Patel AR, Singh R, Sapsford RJ, et al. Human rhinovirus infection during naturally occurring COPD exacerbations. The European respiratory journal. [Research Support, Non-U.S. Gov't]. 2014 Jul;44(1):87-96.

E11. Barker BL, Haldar K, Patel H, Pavord ID, Barer MR, Brightling CE, et al. Association between pathogens detected using quantitative polymerase chain reaction with airway inflammation in COPD at stable state and exacerbations. Chest. [Research Support, Non-U.S. Gov't]. 2015 Jan;147(1):46-55.

E12. Singh R, Mackay AJ, Patel AR, Garcha DS, Kowlessar BS, Brill SE, et al. Inflammatory thresholds and the species-specific effects of colonising bacteria in stable chronic obstructive pulmonary disease. Respiratory research. [Research Support, Non-U.S. Gov't]. 2014;15:114.
