# Supplementary material for: Blood and sputum eosinophils in COPD; relationship with bacterial load
Source: Respir Res. 2017 May 8;18:88. doi: 10.1186/s12931-017-0570-5 (PMC5422866; doi:10.1186/s12931-017-0570-5)
Supplement: Additional file 2: Figure S1. — Outline of the methods used for the processing of sputum qPCR and differential cell count samples. (PPTX 64.1 kb) [file 12931_2017_570_MOESM2_ESM.pptx]

## Slide 1
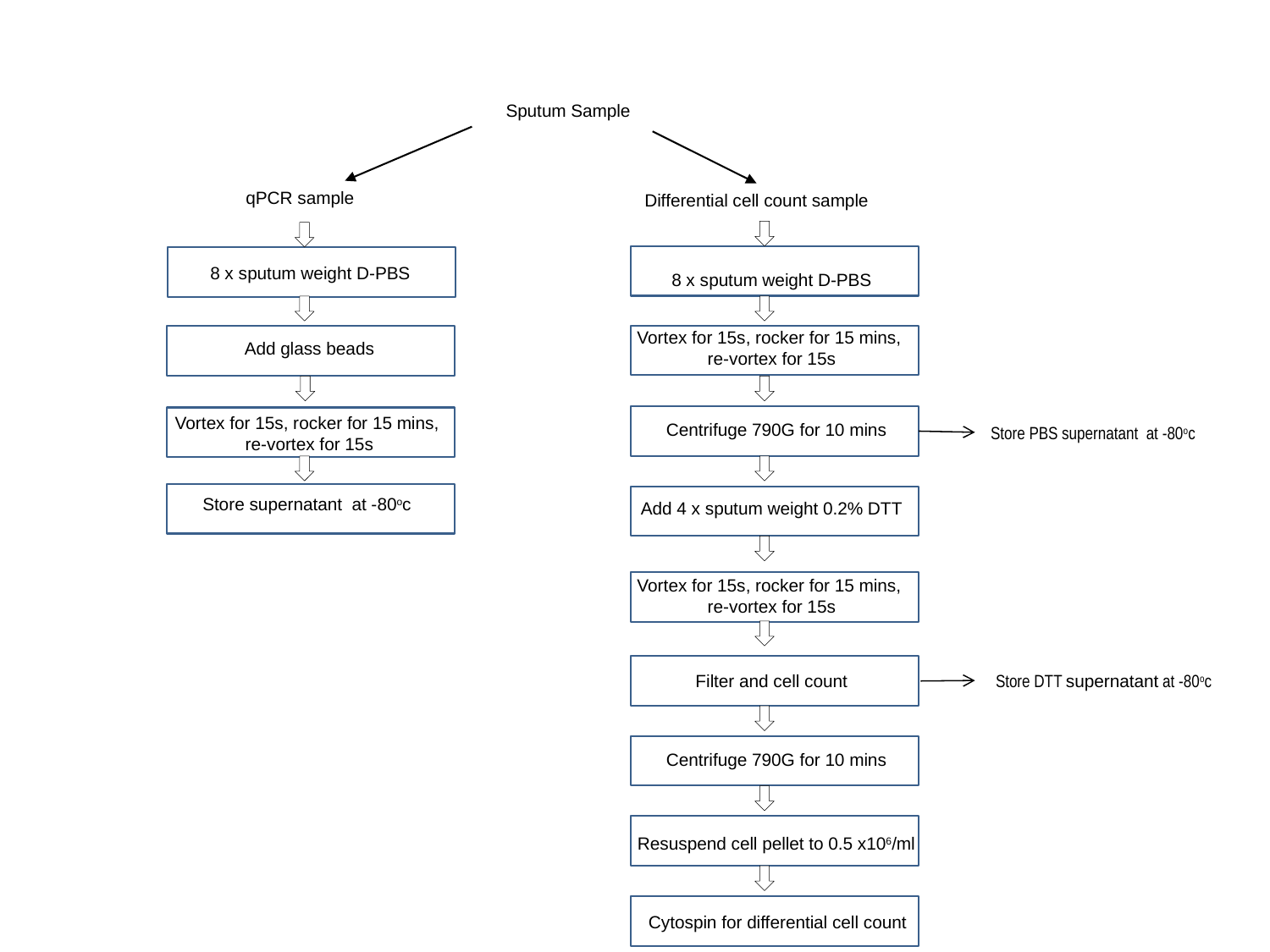

Sputum Sample
qPCR sample
Differential cell count sample
8 x sputum weight D-PBS
8 x sputum weight D-PBS
Vortex for 15s, rocker for 15 mins,
re-vortex for 15s
Add glass beads
Vortex for 15s, rocker for 15 mins,
re-vortex for 15s
Centrifuge 790G for 10 mins
 Store PBS supernatant at -80oc
 Store supernatant at -80oc
Add 4 x sputum weight 0.2% DTT
Vortex for 15s, rocker for 15 mins,
re-vortex for 15s
Filter and cell count
Store DTT supernatant at -80oc
Centrifuge 790G for 10 mins
Resuspend cell pellet to 0.5 x10^6^/ml
Cytospin for differential cell count
