# Supplementary material for: Blood and sputum eosinophils in COPD; relationship with bacterial load
Source: Respir Res. 2017 May 8;18:88. doi: 10.1186/s12931-017-0570-5 (PMC5422866; doi:10.1186/s12931-017-0570-5)
Supplement: Additional file 3: Figure S2. — A summary of the number of patients with blood / sputum eosinophil counts and qPCR data at exacerbation alone, or coupled to baseline measurements. PPM=potentially pathogenic microorganisms. (PPTX 51.6 kb) [file 12931_2017_570_MOESM3_ESM.pptx]

## Slide 1
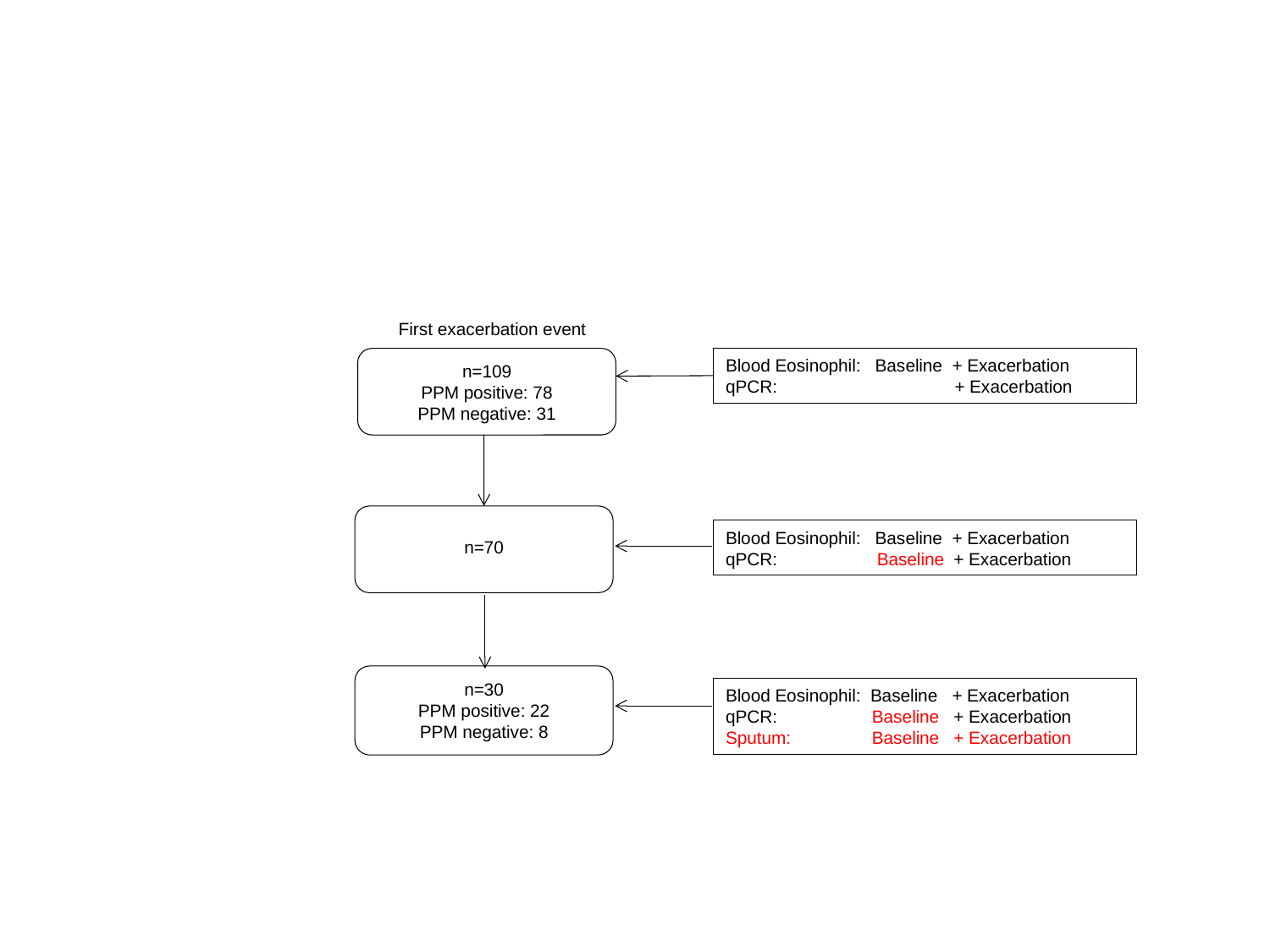

First exacerbation event
n=109
PPM positive: 78
PPM negative: 31
Blood Eosinophil: Baseline + Exacerbation
qPCR:	 + Exacerbation
n=70
Blood Eosinophil: Baseline + Exacerbation
qPCR:	 Baseline + Exacerbation
n=30
PPM positive: 22
PPM negative: 8
Blood Eosinophil: Baseline + Exacerbation
qPCR:	 Baseline + Exacerbation
Sputum:	 Baseline + Exacerbation
