# Supplementary figures and images for: Blood and sputum eosinophils in COPD; relationship with bacterial load
Source: Respir Res. 2017 May 8;18:88. doi: 10.1186/s12931-017-0570-5 (PMC5422866; doi:10.1186/s12931-017-0570-5)

## Slide 1
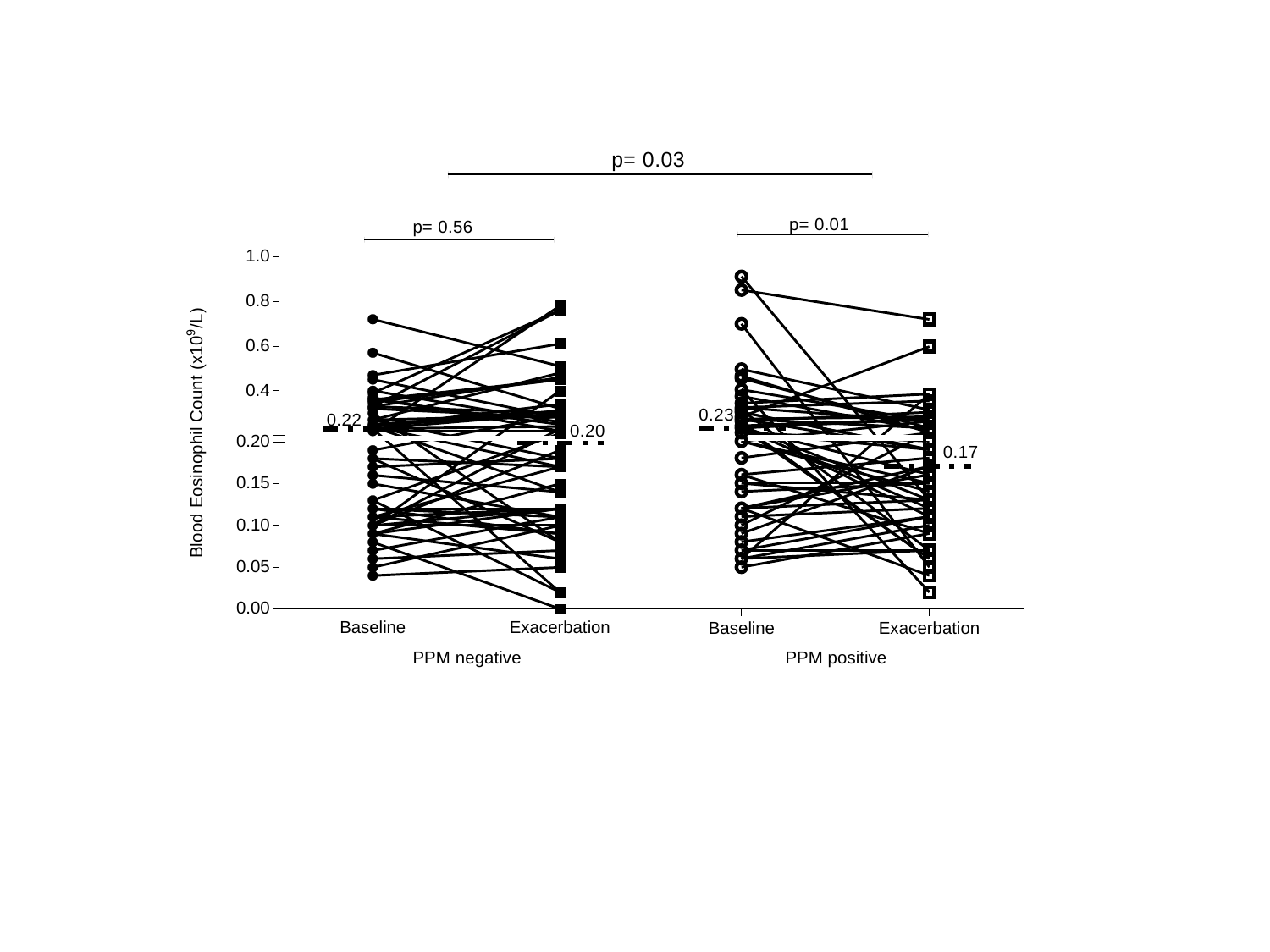

Supplement: Additional file 4: Figure S3. — The blood eosinophil count at baseline and exacerbation in patients defined as PPM positive or PPM negative at exacerbation (PPM threshold value of 1x10 ). PPM=potentially pathogenic microorganisms. Dotted lines represent median values. (PPTX 274 kb) [file 12931_2017_570_MOESM4_ESM.pptx]

## Slide 1
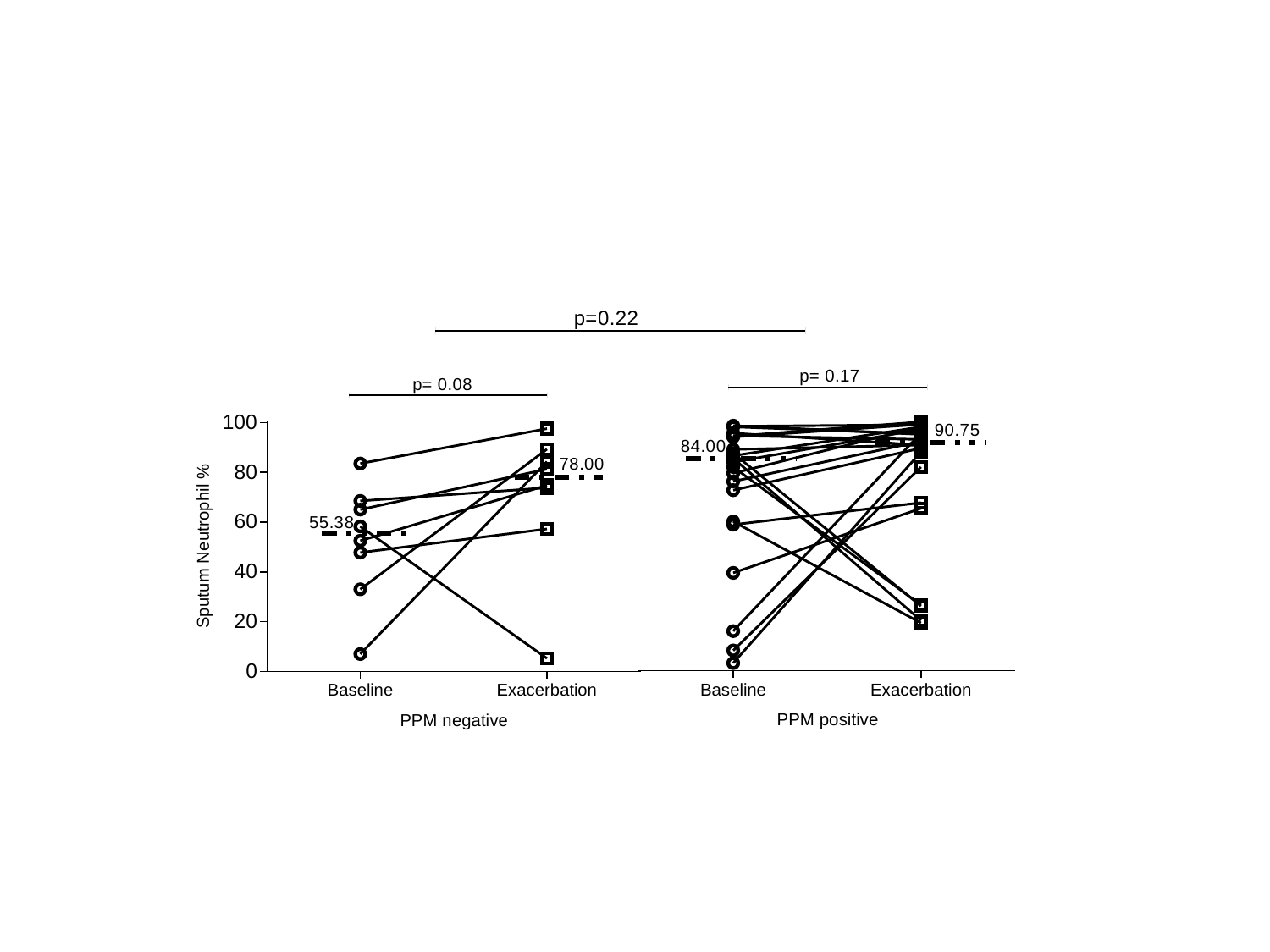

Supplement: Additional file 6: Figure S4. — The sputum neutrophil % at baseline and exacerbation in patients defined as PPM positive or PPM negative at exacerbation. PPM=potentially pathogenic microorganisms. Dotted lines represent median values. (PPTX 189 kb) [file 12931_2017_570_MOESM6_ESM.pptx]
