# Supplementary material for: Blood and sputum eosinophils in COPD; relationship with bacterial load
Source: Respir Res. 2017 May 8;18:88. doi: 10.1186/s12931-017-0570-5 (PMC5422866; doi:10.1186/s12931-017-0570-5)
Supplement: Additional file 5: Table S1. — The change in blood eosinophil count between RV negative and RV positive exacerbations. (PPTX 18.8 kb) [file 12931_2017_570_MOESM5_ESM.pptx]

## Slide 1
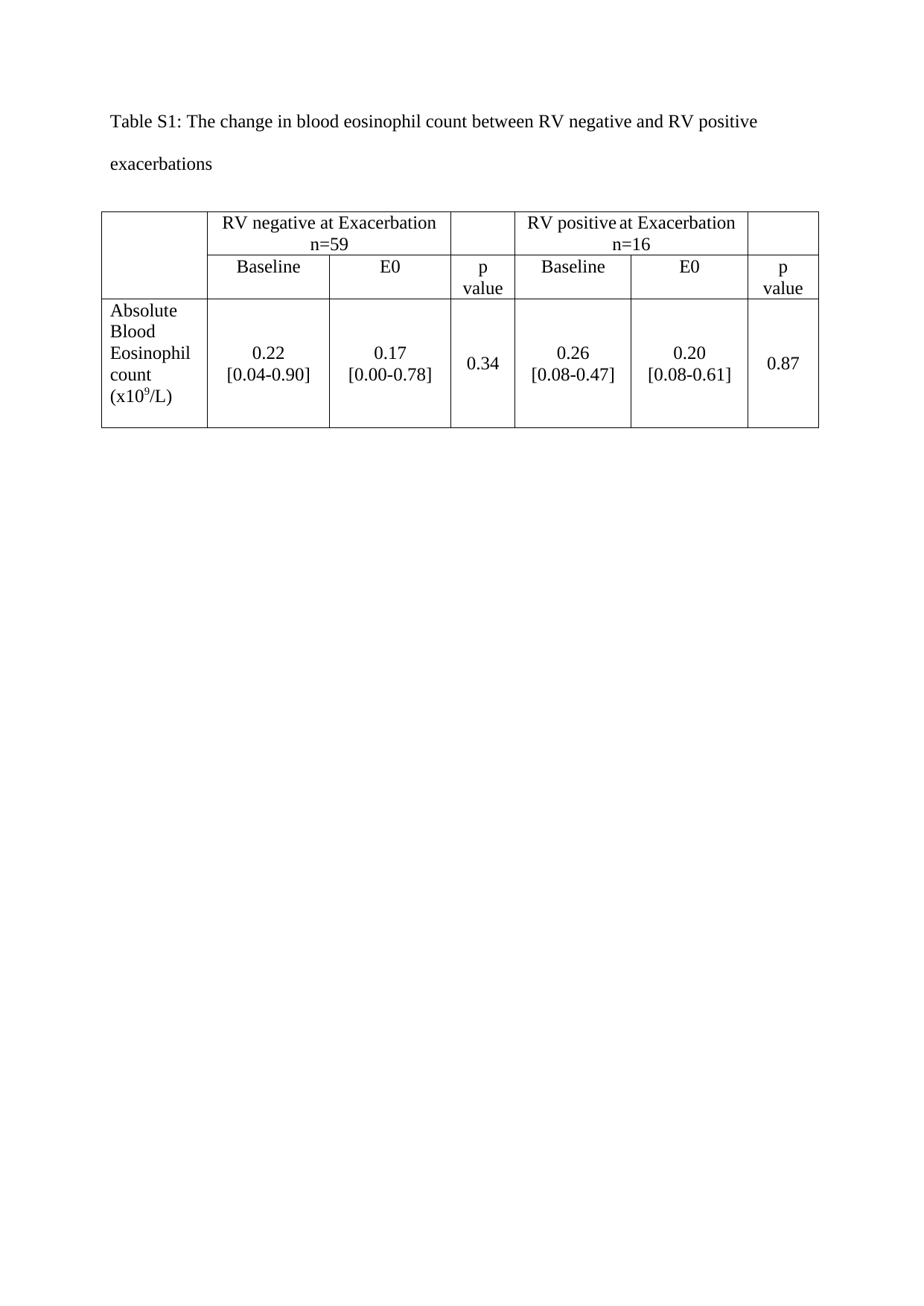

Table S1: The change in blood eosinophil count between RV negative and RV positive exacerbations
| | | | | | | |
| --- | --- | --- | --- | --- | --- | --- |
| | RV negative at Exacerbation n=59 | | | RV positiveat Exacerbation n=16 | | |
| Baseline | E0 | p value | Baseline | E0 | p value |
| Absolute Blood Eosinophil count (x109/L) | 0.22 [0.04-0.90] | 0.17 [0.00-0.78] | 0.34 | 0.26 [0.08-0.47] | 0.20 [0.08-0.61] | 0.87 |
